# Influence of social network on drug use among clients of methadone maintenance treatment centers in Kunming, China

**DOI:** 10.1371/journal.pone.0200105

**Published:** 2018-07-03

**Authors:** Ling Shen, Sawitri Assanangkornchai, Wei Liu, Le Cai, Fei Li, Songyuan Tang, Jiucheng Shen, Edward B. McNeil, Virasakdi Chongsuvivatwong

**Affiliations:** 1 Epidemiology Unit, Faculty of Medicine, Prince of Songkla University, Hat Yai, Thailand; 2 School of Public Health, Kunming Medical University, Kunming, China; 3 School of Basic Medical Science, Kunming Medical University, Kunming, China; 4 Yunnan Institute for Drug Abuse, Xi Shan District, Kunming, China; China Medical University, TAIWAN

## Abstract

**Aims:**

To examine drug use behavior of clients attending Methadone Maintenance Treatment (MMT) programs and its relationship with the clients’ social network characteristics.

**Design:**

Cross-sectional study.

**Setting:**

Four MMT clinics in Kunming, Yunnan province, China.

**Participants:**

324 consecutive MMT clients.

**Measurements:**

A structured, self-completed questionnaire on background characteristics and existing social network. Current drug use was assessed by urine test for opiate metabolites.

**Analysis:**

The association between client's social network characteristics and their own current drug use behavior is analysed using multiple logistic regression adjusting for socio-demographic characteristics. Adjusted odds ratios (AOR) with 95% confidence intervals (CI) are obtained to give the strength of the associations.

**Findings:**

MMT clients were more likely to concurrently use heroin while attending MMT if their social network had any of the following characteristics: more than half of the members were older than them (AOR = 1.03, 95% CI = 1.00,1.06), any member had a high level of influence on them (AOR = 6.47, 95% CI = 2.86,14.65) and any member joined them in using drugs (AOR = 1.94, 95% CI = 1.04,3.63). Having a social network member who could provide emotional support (AOR = 0.11, 95% CI = 0.03,0.35), having a spouse and/or child in their social network (AOR = 0.44, 95% CI = 0.24,0.81) and having a social network member with a high level of closeness (AOR = 0.28, 95% CI = 0.09,0.90) were associated with a decreased odds of heroin use.

**Conclusion:**

Social networks who could provide MMT clients with emotional support and a close relationship were significant factors for reducing the risk of concurrent drug use among clients attending MMT clinics in Kunming, China. Behavioral interventions should address the role of family and social network members in providing support to these clients.

## Introduction

### Significance of methadone maintenance treatment

In the last 20 years, injection drug use (IDU), especially involving heroin, has played an important role in the rapid spread of HIV/AIDS in China [1]. By the end of 2014, over 500 thousand people were living with HIV/AIDS and between 1989 and 2014 159,000 people died from HIV/AIDS. The percentage of new cases attributed to IDU transmission decreased from 44.2% in 2005 to 6.0% in 2014 [2]. To deal with the severe IDU-driven HIV epidemic, methadone maintenance treatment (MMT) programs were established in 8 clinics in 2004 around the country. By the end of 2014, 767 clinics were operational administering treatment to about 184,000 clients [2]. Prior to 2010, in Yunnan province of southwest China, a province that contains 25% of all HIV cases in China, 13 MMT clinics treated a total of 7,241 registered clients [3]. By the end of 2014, there were 68 MMT clinics treating 39,472 registered clients [4, 5]. In China, every client pays 10 yuan (US $1.50) for one dose of methadone per day under the supervision of the MMT clinic staff. Clients are required to take a urine test in the clinic one random day per month. The clinics provide comprehensive services, including psychological counseling, health education, and treatment referral [6]. Growing evidence shows that MMT has the effect of reducing HIV-risk behaviors and drug-related crime, improving social happiness and well-being, and increasing quality of life and chance of employment [7–10].

### Concurrent drug use

Many MMT clients continue to engage in drug-using behaviors during treatment, resulting in high drop-out rates [11–13]. Traces of opiates have been found in several urine samples of MMT clients [11, 14–16]. Many studies and program evaluations have revealed factors associated with concurrent drug use among MMT clients. For example, higher doses of methadone, appropriate psychological counseling, and comprehensive services were found to be associated with less illicit opiate abuse and longer retention in treatment [17–19]. However, these studies examined only individual factors; only few included interpersonal network factors[20].

### Social networks

Social networks play a crucial role in generating and disseminating social influence [21]. Social network analysis is a useful tool to study the social context of HIV/AIDS risk behaviors among injection drug users [22]. A social network includes three components: social network integration, network structure, and relationship content [23, 24]. Social network integration describes the type of relationship between the members in the network, such as intimate partners, work colleagues, and drinking friends, as well as the level of trust and closeness between them. Social network structure illuminates the relationships between one person and other network members (for example, size and density of network) [22, 25]. The relationship content means the function of the relationship, which is distinguished in terms of source or social roles, for example spouse, friend and co-worker, and domain of support, for example instrumental, emotional, and informational [22]. Social network functions include social support and social norms that affect network members’ adoption or maintenance of behaviors [26, 27]. Social support includes forms which cover tangible or emotional support [28] and has been reported to be associated with a decreased risk of HIV risk behavior [29–33]. Given that limited studies have been conducted on social networks among MMT clients, especially in Kunming, the capital city of Yunnan province, an area in the southwest of China where HIV/AIDS transmission is a growing concern [5], the present study characterizes MMT clients’ social network and describes its relationship with current drug use behaviors. Understanding the characteristics of social networks of MMT clients and how they affect drug use behaviors will be useful for provincial governments to tailor strategies and preventive interventions to those with HIV/AIDS [22, 34–38].

## Materials and methods

### Study site and subjects

This cross-sectional survey was conducted in Kunming city, Yunnan province, China. A total of 324 participants were consecutively recruited from four out of 11 randomly sampled MMT clinics in 2016. The four selected clinics treat over 200 clients per day and are distributed in different parts of the city. To be eligible to participate in an MMT program in China, a person must: 1) have made several failed attempts at maintaining abstinence, 2) be at least 20 years of age, 3) be a local resident of the area and be able to prove permanent residency, and 4) have no history of causing or participating in civil unrest.

All study documents and procedures were approved by the institutional review boards of the Faculty of Medicine, Prince of Songkla University (59-153-18-5) and Kunming Medical University. Anonymity of the data was assured to the participants after they were given detailed information of the study procedures and before they signed the informed consent form.

### Data collection and measures

Five well-trained research assistants interviewed MMT clients face-to-face in private rooms of the participating clinics. The medical staff at the clinics assessed the client’s clinical condition before referring them to the research assistants. Clients who were cognitively impaired, intoxicated, or suffering from withdrawal symptoms were not referred to the interview. The informed consent process was thus undertaken only when the client was in full capacity. The interviews took about 50 minutes to complete. Demographic information collected included age, gender, religion, marital status, education, ethnic group, occupation, and current employment. Use of alcohol, opium, heroin, morphine and other drugs in the past 30 days were also collected.

Data on social support networks were collected with a set of questions enquiring about the MMT client’s relationships with people in his/her own social network. First, the index client identified all people whom he/she knew very well and likewise who knew him/her very well. The lists of identified people were then narrowed down to those who had contact with the client either through face-to-face meeting, telephone, or any other channel within the past three months. From this list, each client then identified the five closest people in their network. Detailed characteristics of each identified person were elicited, including nickname, relationship with the client, age, sex and other demographic characteristics, types of support and influence on the client, and risk behaviors relating to drug use and sexual contact with the client. Due to confidentiality issues, the ties among members in the same network could not be identified. The network density, defined as the actual number of ties among all individuals in a social network divided by the number of possible ties [39], was thus not calculated.

Level of closeness for each of the five persons identified by the participants was measured with the following question: “How close do you feel to this person?” The response, measured on a 4-point scale, ranged from not close at all (1), a little close (2), close (3), and very close (4). Level of influence was measured with the following question: “Which level does he/she influence you?” The response, measured on a 5-point scale, ranged from not at all (1) to highly influential (5). Joining in drug use was measured with the following question: “Does he/she sometimes join you while using drugs?”, with response “Yes” or “No”. Emotional support was measured with the following question: “If you want to talk to someone about things that are very personal and private, would you talk to this person?”, with response “Yes” or “No”. Financial support was measured with the following question: “If you needed to borrow 100 yuan, would this person lend or give you this amount of money?”, with response “Yes” or “No”. Health advice was measured with the following question: “If you needed advice for health problems, would this person give you this advice?”, with response “Yes” or “No”.

After the interview, a urine specimen was collected and tested immediately for opiate metabolites by colloidal gold rapid detection method using the One Step Test Strips (Abon Biopharma, Co, Ltd, Hangzhou, China). If a participant had a positive urine test result, he or she was considered as a current heroin user.

### Statistical analysis

All data analyses were performed using the R language and environment (Version 3.3.4). Descriptive analyses were performed to describe socio-demographic characteristics, current drug use behaviors, and characteristics of social network among the MMT clients. Size of the social network was described as a number of members in the network. We recognized that the client’s characteristics determine how much he/she was connected with his/her social network. Each network variable was described in relation to the indexed client’s characteristics, using the median for discrete variables. For example, an MMT client with an older-member network was defined as a client whose social network members’ median age was higher than him/herself and a different-sex network as having >50% of the members of the opposite sex from the indexed MMT client. Regarding social network integration variables, including levels of closeness and influence, we assumed that if any of the members of the network provided a high level (level 3 for closeness and level 4 or 5 for influence), that network was defined as having a high level of closeness or influence, respectively. Likewise, for social network function variables, if any of the network members were perceived as being a possible source of financial or emotional support, or health advice, that network was labeled as having that function. Univariate and logistic regression modeling was used to examine the effect of social network factors on heroin use based on the urine test result. Demographic characteristics, including age, gender, education, current employment status and family size of the MMT clients were adjusted for in the multivariate models. Records containing less than 1% of missing values in any variable were excluded from the analysis. Variables having a p-value less than 0.05 were considered as significant predictors in the final model.

## Results

### Socio-demographic characteristics of MMT clients

Table 1 shows the distribution of socio-demographic characteristics of the study participants. The mean (standard deviation) age of the sample was 45.2 (5.9) years. The majority were males (76.9%) of Han ethnicity (83.0%), had no religion (66.4%), married (64.8%), and high-school educated (61.7%). Most (40.7%) had full-time employment but a sizeable number were unemployed (36.4%). The distribution of the participant's characteristics was similar among those attending Clinics 1–3. Participants attending Clinic 4, a clinic in a suburb far from the city center, were predominantly male, less educated, single, belonged to an ethnic minority group, and employed in full-time work.

**Table 1:** Distribution of sociodemographic variables of subjects by clinic; N (%).

	Clinic 1	Clinic 2	Clinic 3	Clinic 4	Total
Total					324 (100.0)
Age					
Mean (SD)	46.2 (5.9)	44.3 (6.2)	44.7 (5.7)	45.5 (7.2)	45.2 (6.3)
Gender					
Male	60 (74.1)	58 (71.6)	57 (70.4)	74 (91.4)	249 (76.9)
Female	21 (25.9)	23 (28.4)	24 (29.6)	7 (8.6)	75 (23.1)
Religion					
Buddhist	11 (13.6)	19 (23.5)	16 (19.8)	19 (23.5)	65 (20.1)
Christian or Muslim	12 (14.8)	12 (14.8)	10 (12.3)	10 (12.3)	44 (13.5)
None	58 (71.6)	50 (61.7)	55 (67.9)	52 (64.2)	215 (66.4)
Education					
No formal education	5 (6.2)	3 (3.7)	9 (11.1)	17 (21.0)	34 (10.5)
Primary school only	10 (12.3)	25 (30.9)	23 (28.4)	32 (39.5)	90 (27.8)
High school and above	66 (81.5)	53 (65.4)	49 (60.5)	32 (39.5)	200 (61.7)
Marital status					
Single	13 (16.0)	5 (6.2)	6 (7.4)	17 (21.0)	41 (12.7)
Married	47 (58.0)	55 (67.9)	61 (75.3)	47 (58.0)	210 (64.8)
Other^*^	21 (26.0)	21 (25.9)	14 (17.3)	17 (21.0)	73 (22.5)
Ethnic group					
Han	71 (87.7)	67 (82.7)	68 (84.0)	63 (77.8)	269 (83.0)
Other	10 (12.3)	14 (17.3)	13 (16.0)	18 (22.2)	55 (17.0)
Employment status					
Unemployed	29 (35.8)	37 (45.7)	39 (48.1)	13 (16.0)	118 (36.4)
Part-time	14 (17.3)	10 (12.3)	10 (12.3)	14 (17.3)	48 (14.8)
Full-time	32 (39.5)	26 (32.1)	25 (30.9)	49 (60.5)	132 (40.7)
Self-employed	4 (4.9)	7 (8.6)	5 (6.2)	3 (3.7)	19 (5.9)
Other	2 (2.5)	1 (1.2)	2 (2.5)	2 (2.5)	7 (2.2)

^*^Separated/divorced/widowed

### Concurrent drug use behaviors of MMT clients

Self-reporting of injection drug use in the past 30 days was low with 26 (8.0%) using heroin and three (1.0%) using morphine. Twenty (6.2%) participants reported smoking or sniffing opium, morphine, or heroin. More than half of the participants (166, 51.2%) reported drinking alcohol in the past 30 days. Six participants (1.9%) smoked cannabis, 12 (3.7%) smoked crystal methamphetamine (ice), and seven (2.3%) reported taking oral 3,4-methylenedioxymethamphetamine (ecstasy) pills. All participants gave urine samples, of which 84 (25.9%) tested positive for morphine or heroin.

### Characteristics of social network

Table 2 shows the social network characteristics of study participants. Regarding network structure, 324 MMT clients reported having 1,275 members in their social network (mean = 3.9), while 41.4% and 32.4% had an older and different-sex network, respectively. Regarding relationship with the indexed clients, about half (54.0%) had parents in their social network, and one-third included friends (33.3%) and their spouse (32.7%). Regarding the degree of closeness and influence between the clients and their social networks, 92.0% of the participants had at least one very close member, 67.0% had at least one very influential member and 24.4% had at least one member in their network joining them in using drugs. The majority perceived that they knew at least one member who would provide financial (84.9%), or emotional (87.7%) support while less than half (45.1%) perceived that they could ask someone in their network for health advice.

**Table 2:** Characteristics of social network of MMT clients (N = 324).

Network characteristic	n (%)
**Structure**	
Size (mean ± SD)	3.9 ± 1.0
Older (median age of members > client’s age)	134 (41.4)
Different sex (>50% of members of opposite sex)	105 (32.4)
**Relationship with the indexed client**	
Has parents in the network	175 (54.0)
Has friends in the network	108 (33.3)
Has a spouse in the network	106 (32.7)
Has children in the network	92 (28.4)
Has relatives in the network	65 (20.1)
Has colleague in the network	32 (9.9)
Has at least one MMT friend in the network	15 (4.6)
**Closeness and influence**	
At least one very close member in the network	298 (92.0)
At least one very influential member in the network	217 (67.0)
At least one member of the network joining drug use	79 (24.4)
Perceived type of support^*^	
Financial	275 (84.9)
Emotional	284 (87.7)
Health advice	146 (45.1)

SD = standard deviation.

*At least one member of the network.

### Relationship between social network and drug use

Social network variables significantly associated with a positive urine test result included age of the members (older/younger), perceived ability to provide emotional support, having a spouse and/or child in the network, level of closeness, and level of influence. Clients whose network members were mostly older than themselves were 1.03 times more likely to use heroin, compared to those with younger or similar aged members. Having at least one member of the network who could provide emotional support was a protective factor for heroin use. Having a spouse and/or child in the network and having at least one very close member were also protective factors for heroin use. Finally, having at least one highly influential member in the network and one who joined them using drugs increased the risk for heroin use (Table 3).

**Table 3:** Regression results of social network factors associated with result of urine test among MMT clinics in Kunming in Yunnan Provence, China (logistic regression predicting urine test: Positive vs. negative).

Characteristic of the network	Crude OR (95% CI)	Adjusted OR (95% CI)^†^
**Significant risk factors**		
Highly influential: yes vs. no	5.75 (2.75,12.03)	6.47 (2.86,14.65)*
Joining in drug use: yes vs. no	2.73 (1.59,4.7)	1.94 (1.04,3.63)*
Age: older vs. same age or younger	1.03 (1.00,1.05)	1.03 (1.00,1.06)*
**Significant protective factors**		
Could provide emotional support: yes vs. no	0.26 (0.13,0.51)	0.11 (0.03,0.35)*
Spouse and/or child in network: yes vs. no	0.51 (0.3,0.85)	0.44 (0.24,0.81)*
Close relationship: yes vs. no	0.64 (0.27,1.49)	0.28 (0.09,0.9)*
**Non-significant network factors**		
Financial support: yes vs. no	0.49 (0.26,0.93)	1.81 (0.63,5.22)
Size of network (continuous variable)	0.99 (0.82,1.18)	1.17 (0.89,1.53)
Health advice: yes vs. no	0.73 (0.44,1.21)	0.85 (0.47,1.56)

^**†**^Adjusted for demographic characteristics: age, gender, education, current employment, number of children, and cohabiting with network members.

^*^P-value < 0.05

## Discussion

Concurrent heroin use among MMT clients continues to be a global issue [11]. Our paper reports the significant associations between various social network factors and heroin use during MMT attendance in Kunming city, Yunnan province, China. This paper not only supports previous findings regarding factors influencing concurrent injection drug use among MMT clients but also provides new data on structure and function of social networks of the MMT clients.

In this study, about one quarter of the MMT clients tested positive for opiates based on their urine sample, a result consistent with previous studies [12, 13]. However, there was a high discordance between self-reported heroin use and the positive urine test result (8.0% vs. 25.9%), a finding similar to another study [12, 13]. Li and colleagues [11] showed that 44.9% of MMT clients in China either reported illicit heroin use over the past 30 days or had a positive urine test. As injection drug use is an illegal behavior in most countries, users are unlikely to disclose their drug use behaviors, especially when they are attending a MMT clinic. The self-reporting of drug use is thus often underestimated.

In the last decade, the abuse of amphetamine-type stimulants (ATS) including crystal methamphetamine (ice) and methylenedioxymethamphetamine (ecstasy) has been growing fast in China. The use of such drugs is also common among MMT clients [40]. As seen in our study, about 6% of the participants used ice or ecstasy in the past 30 days despite their active attendance at a MMT clinic. The reasons for MMT clients using non-opiate drugs concurrently may be that the combination of methamphetamine and methadone produce greater effects than either drug alone by increasing pleasurable feelings [41, 42]. It might also be due to the unavailability of heroin and thus any drug within their reach could be substituted [43, 44]

Our findings indicate multiple significant associations between an individual’s drug using behavior and characteristics of his/her social network. These associations suggest multiple ways in which social networks might influence risky behaviors of their members.

Our results suggest that clients who had a spouse and/or child in the social network were significantly less likely to use drugs while attending a MMT clinic. This finding highlights the role of family members in preventing drug use among MMT clients. Having a spouse or child in the network may provide emotional and other types of support, which can reduce stress and pressure and subsequently lead to decreased drug use. This is supported by the fact that the clients who perceived that they had emotional support in their network were significantly less likely to use drugs. This finding has also been seen with HIV/AIDS risk behaviors, where social support factors, such as having a high number of people to talk with when being upset, decreased the odds of risky drug use behaviors [30, 31]. Family relationship plays a crucial role in drug recovery, and having a harmonious relationship could potentially encourage treatment participation and compliance [45–47]. These factors are consistent with other studies during treatment [33, 48–50]. Furthermore, our study indicates that perceived emotional support was more powerful than financial support on influencing risky behavior in the participants’ network.

We also found that having a close relationship with the network members was associated with a lower odds of using heroin. However, this finding is inconsistent with previous studies which identified drug network characteristics, such as close ties to others in the network, as being associated with high-risk injection practices [51–56]. This inconsistency may have occurred because most close participants are family members and non-drug using friends who can offer emotional support and bring more communications and interaction in their social support network.

On the contrary, participants who associated with older members and had a highly influential member in the network were more likely to use heroin while attending MMT. These findings have not been reported in other published studies. In fact, MMT clients often suffer from discrimination and stress by the society because of their socially unacceptable behavior. This may lead to smaller peer groups, relatively constant friends, and a relatively closed communication network. Most friends may know each other better in the closed net, including their drug use prior to MMT. Additionally, due to belief of traditional Chinese culture in general, elders have more responsibility by virtue of having more control and more authority in their groups, as well as exerting more impact on young members[45]. Lastly, another risk factor of heroin use found in this study was having at least one network member joining them in using drugs. This finding is consistent with previous studies of injecting partnerships between younger and older injection drug users where the elder users can also link with young users to high-risk injecting environments [21, 57]. Members who joined in drug use behaviors were the key persons for prevention of concurrent drug use among MMT clients. Further research exploring more information about these factors to reduce drug use in MMT programs is needed.

There are several limitations in this study. Firstly, this was a cross-sectional survey and thus we were not able to make any causal inferences. Secondly, we used self-reported data for the number of social networks and characteristics of members in the client's network, thus social desirability and recall biases might be present. Thirdly, urine tests can only identify heroin use within the past 7 days, a fact which might underestimate the actual prevalence of heroin use among our participants. Fourthly, the functional relationship of the social network member in terms of emotional, social, and financial support, closeness, and level of influence was measured using a single question for each of these structures. However, during the pretesting stage, it was noticed that the initial version of the questionnaire was too long, causing many MMT clients to become weary. In the main study, we thus reduced the length of the questionnaire by selected only the most important items, that is, those which could best reflect the parameters that they were intended to measure. Finally, recruitment of participants was restricted to Kunming city, thus the observed prevalence of drug use found in our study may not be generalizable to MMT clients in other areas.

## Conclusions

Due to the negative role played by concurrent drug use by clients attending MMT programs, preventing this illegal behavior may be particularly important in curbing the spread of HIV/AIDS and promoting MMT programs throughout China. Our study was designed to identify concurrent drug use problems among MMT clients that desperately needs to be solved. The study identified associations between concurrent drug use behavior and private social network characteristics of MMT clients. Moreover, a notable finding was that some variables in the social network such as having a spouse and/or child in their social network, having members who can be relied on to provide emotional support, and having close members, were protective factors for concurrent heroin use in MMT clients. On the other hand, significant risk factors such as belonging to a predominantly older and highly influential social network, and using drugs with others, were also found. These findings highlight the complexity of concurrent drug use behaviors among MMT clients and underscore the importance of increasing emotional support in social networks in order to reduce drug use in the community.

Behavioral interventions should mention the role of family and social network members in providing support to the MMT clients. Health education for clients in MMT clinics should be supplemented with emotional module lessons and skills on how to avoid succumbing to peer pressure from friends who use drugs.

## Supporting information

S1 Dataset (MMTD 0423d.sav). (SAV)

S1 Table P-values for Wald’s tests and likelihood ratio tests for the logistic regression modeling of social network factors associated with result of urine test among MMT clinics in Kunming in Yunnan Provence, China. (CSV)
